# Fluorescence recovery in the super-resolution regime reveals subcompartments of 53BP1 foci

**DOI:** 10.1016/j.crmeth.2025.101118

**Published:** 2025-08-04

**Authors:** Chengchen Wu, Janeth Catalina Manjarrez-González, Muntaqa Choudhury, Noor Shamkhi, Siwen Ding, Vishnu M. Nair, Viji M. Draviam

**Affiliations:** 1 Centre for Cell Dynamics, Centre for Molecular Cell Biology, School of Biological and Behavioural Sciences, Queen Mary University of London, London, UK

**Keywords:** structured illumination microscopy, DNA damage repair dynamics, FRAP and super-resolution, 53BP1, DNA repair subcompartments, DNA double-strand break (DSB) and long-term live-cell imaging

## Abstract

Double-strand break (DSB) repair protein 53BP1 (p53 binding protein-1) mediates long-range DNA end-joining and heterochromatin maintenance. We exploit lattice structured illumination microscopy (SIM) (dual iterative SIM [*di*SIM, also called SIM^2^]; ∼60 nm lateral resolution) alongside lattice light-sheet microscopy and fluorescence recovery after photobleaching (FRAP) and reveal differences in 53BP1 foci contour and composition. Compact 53BP1 foci remain stationary, while amorphous foci undergo dynamic shape changes. Using FRAP in the super-resolution (SR) regime (FRAP-SR), we show that amorphous 53BP1 foci recover 53BP1-EGFP signals rapidly exhibiting subcompartments, indicating differential protein mobilities and functions within a single foci. In contrast, compact foci recover 53BP1-EGFP uniformly as a single compartment but show heterogeneous recovery rates. In cells released from a DNA replication block, 53BP1-EGFP shows increased mobility in amorphous foci compared to compact foci. We discuss the conceptual implications of different 53BP1 mobilities and foci contours and how the FRAP-SR method transforms studies of dynamic 60–100 nm structures.

## Highlights

• Super-resolution imaging with *di*SIM/SIM^2^ can achieve a lateral resolution of 60 nm
• Super-resolved 53BP1 foci display two distinct contours: amorphous and compact
• Amorphous 53BP1-GFP foci recovery reveals multiple subcompartments
• Compact 53BP1-GFP foci recovery is uniform, with heterogeneous rates across cells

## Motivation

Live studies of the DNA damage repair process are highly sensitive to phototoxicity. We introduce fluorescence recovery after photobleaching in the super-resolution regime (FRAP-SR) by integrating lattice structured illumination microscopy (dual iterative SIM [*di*SIM, also called SIM^2^]), a gentle live-cell SR method, with lattice light-sheet microscopy and FRAP. FRAP-SR achieves ∼60 nm spatial resolution alongside kinetic measurements of protein interactions. FRAP-SR studies of 53BP1, which forms liquid condensates at DNA double-strand break (DSB) sites, reveal subcompartments within a 53BP1 foci, indicating differences in protein mobilities and functions. FRAP-SR is a powerful approach to dissect dynamic spatial organization and kinetic behavior of protein assemblies at the nanoscale.

## Graphical abstract

**Figure:**
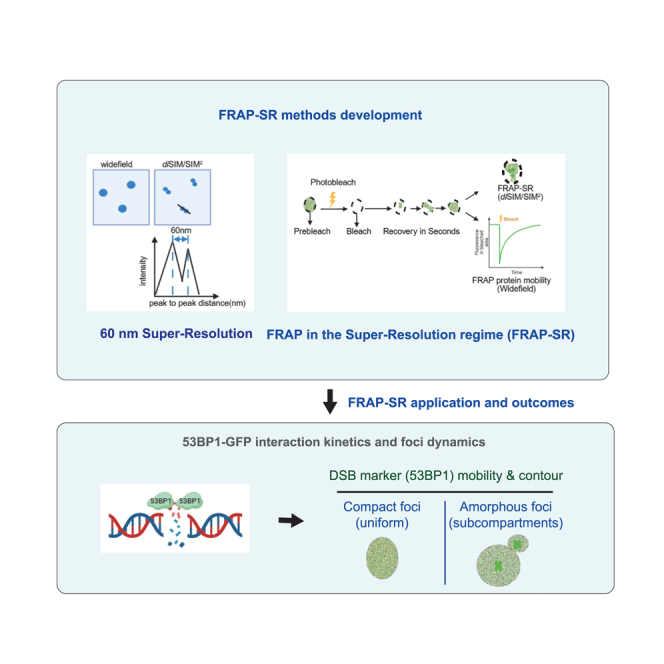

## Teaser

Wu et al. exploit 60 nm lateral resolution of *di*SIM/SIM^2^-processed super-resolution (SR) images to develop FRAP in the SR regime, which reveals distinct types of DNA-damage-repair-associated 53BP1 foci, including those with subcompartments.

## Introduction

Double-strand breaks (DSBs) are highly toxic lesions that, if uncorrected, can cause mutations and chromosomal instability, leading to cancers.^1^ DSBs signal a histone modification cascade recognized by dimers of 53BP1 (tumor protein p53 binding protein-1; TP53BP1), leading to the formation of higher-order 53BP1 oligomers and a mature foci structure.^2^^,^^3^^,^^4^^,^^5^ 53BP1 is a large, 1,972-aa-long protein that forms discrete nuclear foci that enrich downstream checkpoint effectors^6^^,^^7^ within a minute of DNA damage. These foci can resolve within 2 min.^8^ Similar rapid recruitment and release have been observed in other components of the DNA damage response (DDR) pathway,^9^ highlighting 53BP1’s role in a highly dynamic and macromolecular assembly process.

Phase separation of 53BP1 determines the liquid-like behavior of DNA repair compartments.^10^ Beyond DNA repair, 53BP1 regulates the heterochromatin structure through phase separation^11^ and facilitates long-range DNA end-joining interactions^12^^,^^13^ during V(D)J recombination and class-switch recombination. 53BP1 foci can also arise in response to replication stress during the G1 phase following mitosis.^14^^,^^15^ Importantly, 53BP1 foci can remain unresolved for days in multinucleated G1 cells, while laser-damage-induced 53BP1 foci within the same nuclei can resolve within minutes.^8^ Thus, 53BP1 regulates many macromolecular nuclear events, and 53BP1 foci can be stable or dynamic within the same cell.^8^

Various approaches exist to damage DNA and track its repair live using super-resolution (SR) microscopy^16^—for example, SR imaging has revealed the rearrangement of 53BP1 signals during DNA repair.^17^^,^^18^^,^^19^ However, 53BP1 protein mobilities (interaction dynamics) have not been correlated with 53BP1 foci’s architectural changes, as this requires a combination of fluorescence recovery after photobleaching (FRAP) and SR imaging. Single-molecule FRAP, an SR imaging technique used for the nuclear envelope (40 nm dimensions), has significantly expanded our understanding of nuclear wall proteins,^20^^,^^21^ but this method requires continuous exposure for prolonged periods to collect fluorescent signals, making it unsuitable for studying DNA repair, which is a highly photosensitive process. As opposed to this, lattice structured illumination microscopy (dual iterative SIM [*di*SIM, also called SIM^2^]) is a gentle SR imaging approach and can computationally improve the diffraction-limited lateral resolution by at least 2-fold.^22^^,^^23^ Thus, combining FRAP with lattice SIM can help probe 53BP1 protein dynamics in addition to simultaneous visualization of the foci’s architectural changes in the SR regime, but this has not been reported so far.

Using FRAP to capture protein mobility and subcellular changes in the SR regime, we analyze 53BP1 dynamics within nuclear structures at 60 nm resolution. With the FRAP-SR approach, we reveal subcompartments within 53BP1 foci; these subcompartments display faster 53BP1 protein mobility than the others without subcompartments. Based on super-resolved foci contour and FRAP-informed protein mobility data, we find at least two different types of 53BP1 foci: (1) foci that remain compact and recover as a single compartment during the 3-min FRAP period but show high heterogeneity in 53BP1-EGFP recovery rates and (2) foci that show multiple subcompartments during FRAP and display an amorphous contour that is irregular and dynamically shape changing. While the compact foci appear dormant and largely stationary, the amorphous foci are loose and mobile. Using lattice light-sheet movies of cells released from a DNA replication block, we confirm faster recovery of 53BP1 in amorphous foci compared to compact foci. Thus, by exploiting FRAP-SR as a gentle method to correlate protein diffusion with subcellular structural changes in the SR regime, we show evidence for subcompartments within 53BP1 foci and characterize two distinct 53BP1 foci that differ in their activities.

## Results

### FRAP in the SR regime reveals subcompartments within 53BP1 foci

**Figure 1:**
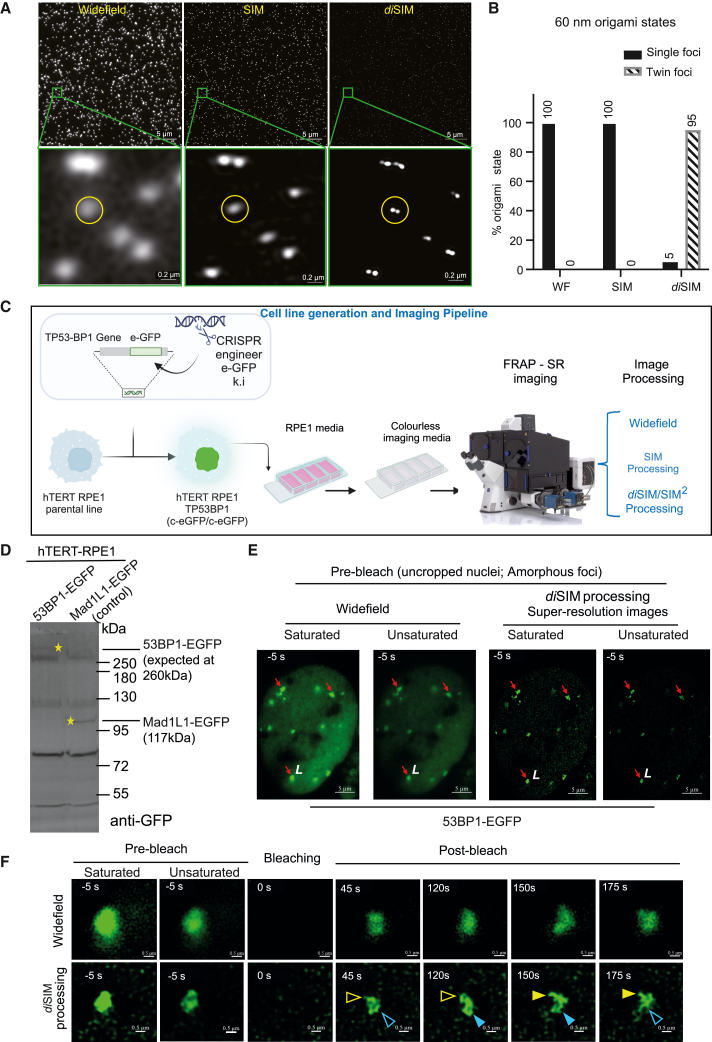
53BP1 foci can be super-resolved into subcompartments using FRAP in the SR regime (A) Uncropped (top) and magnified (bottom) images of 60-nm origami beads imaged and processed for super-resolution using SIM or *di*SIM (dual iterative SIM, also known as SIM^2^) algorithms as indicated. Scale bars as indicated. Green squares in the top row correspond to magnified crops in the bottom row. (B) Graph shows 60-nm origami bead status, either unresolved as a “single foci” or resolved into “twin foci” in unprocessed wide-field (WF) images and super-resolved images (using different processing methods) as indicated. (C) The illustration shows an experimental design to conduct FRAP of 53BP1-EGFP foci in the SR regime. Using CRISPR-Cas9, an in-frame sequence encoding enhanced green fluorescent protein (EGFP) was inserted in the endogenous TP53BP1 loci of the hTERT-RPE1 cell line to introduce a C-terminal EGFP tag (also called eGFP). Cells were grown in RPE1 growth medium (DMEM:F12) and moved to a colorless medium (Leibovitz’s L-15 medium) for live-cell imaging. SIM and FRAP were conducted using a ZEISS Elyra 7 microscope equipped with a Rapp OptoElectronics photomanipulation module. Images were processed with WF reconstruction, and the sum intensity of the z stack was used for FRAP kinetic measurements or SIM- and *di*SIM/SIM^2^-processed for foci structure analysis and size measurements in the SR regime. (D) Immunoblot of lysates of RPE1 53BP1-EGFP or MAD1L1-EGFP (as indicated) probed with anti-GFP antibodies shows the expression of 53BP1-EGFP or Mad1L1-EGFP as expected. RPE1 Mad1L1-EGFP lysate is used as a control. For markers, see a composite pseudo-colored image presented in Figure S1B. (E) Images of an RPE1 53BP1-EGFP nucleus showing 53BP1 foci (red arrows) with an amorphous and irregular contour. WF and *di*SIM-processed images are shown. The letter “L” in white marks the amorphous foci bleached and tracked for recovery in (F). Scale bar as indicated. (F) Cropped time-lapse images of areas marked by a white arrow in (E) show fluorescence intensities before and after bleaching. Recovery images show uneven recovery across the amorphous 53BP1 foci (empty and filled color arrows show the absence and presence of GFP signal intensities, respectively). Saturation levels were set up for post-bleach recovery images (unsaturated pre-bleach images included). Scale bar as indicated. See also Videos S1, S2, and S3.

To demonstrate that we can achieve lateral resolutions of ∼60 nm in our FRAP-SR imaging setup, we imaged 60 nm DNA origami beads and tested two reconstruction algorithms, SIM or *di*SIM (also called SIM^2^), to measure peak-to-peak distances (Figures 1A and 1B). The twin pattern of origami beads separated by 60 nm is fully resolved (Figure 1B) in *di*SIM- but not in SIM-processed images. Measuring the distance between the peak intensities of the twin beads showed a median length of 64.4 nm in 80% of the beads (Figure S1A). We conclude that 60 nm lateral resolution can be achieved using the *di*SIM algorithm for image reconstruction in our FRAP-SR imaging setup.

53BP1-EGFP (also referred as 53BP1-eGFP) foci undergo phase separation^10^^,^^11^ and facilitate long-range interactions between DNA ends.^13^ Cellular levels of 53BP1 can influence the protein’s accumulation on DSB sites. To avoid bias from ectopic 53BP1 protein expression and pleiotropic effects in epithelial cancer cell lines,^24^^,^^25^ we used CRISPR-Cas9 to fluorescently tag endogenous 53BP1 in the hTERT-RPE1 non-transformed cell line (Figure 1C; Table S1). We began with characterizing the EGFP knockin cell line with immunoblotting of RPE1 53BP1-EGFP cell lysates using an anti-GFP antibody, which showed a 260 kDa band corresponding to 53BP1-EGFP—this band was absent in RPE1 Mad1-EGFP cell lysates, which displayed a 117 kDa band corresponding to Mad1-EGFP, as expected (Figures 1D and S1B). Next, using lattice light-sheet microscopy,^26^ we imaged RPE1 53BP1-EGFP cells for 24 h once every 5 min. We confirmed both the steady nuclear levels of 53BP1-EGFP (Video S1) and the typical cell-cycle-regulated pattern of 53BP1 foci disappearing in mitosis (except on kinetochores^27^) and reappearing in G1-phase nuclei as G1 bodies^15^ (Video S2; Figure S1C), demonstrating that 53BP1-EGFP localizes similarly to endogenous 53BP1.

Video S1. Long-term lattice light-sheet imaging shows steady levels of 53BP1 foci in interphase and mitosis, related to Figure 1 Long-term time-lapse images taken once every 5 min of a 4-well glass-bottom ibidi dish from a single position show 53BP1-EGFP foci (scenes) of RPE1 53BP1-EGFP cell line (also called 53BP1-eGFP cell line) showing steady levels of 53BP1-EGFP foci in interphase nuclei.

Video S2. Lattice light-sheet imaging shows cell-cycle-associated appearance and disappearance of 53BP1 foci in interphase and mitosis, respectively, related to Figure 1 Time-lapse images acquired once every 10 min from two distinct positions (scenes) of RPE1 53BP1-EGFP cells show 53BP1-EGFP nuclear signals that disappear during mitosis and reappear in interphase daughter cells. The white arrow marks a mitotic cell, and the orange arrow marks the appearance of mitosis-associated foci.

The wide-field reconstruction of lattice SIM movies of RPE1 TP53BP1-EGFP cells showed that some (but not all) of the 53BP1-EGFP foci presented an amorphous contour that appeared to stretch and collapse loosely (red arrows, Figures 1E; Video S3A). Upon SIM processing, these foci, which we term “amorphous foci”, displayed a dynamic, irregular shape that was not readily evident, although it was present in wide-field unprocessed images (compare Videos S3A–S3C). To further improve SIM outcomes beyond the 120 nm lateral resolution, we employed additional computational image processing methods (*di*SIM, commercial name: SIM^2^) that offered further improvement.^22^ In both SIM- and *di*SIM-processed time-lapse movies, some (but not all) 53BP1-EGFP foci display an amorphous contour and an irregular shape (*n* = 10 cells; Figures 1E and S1D; see Video S3B and S3C for SIM and *di*SIM processing).

Video S3. Series of time-lapse movies of unprocessed or SIM- or diSIM-processed images of RPE1-TP53BP1-EGFP cells, related to Figure 1 A single 53BP1-EGFP foci (marked with red arrow) was photobleached at 5 s. Unprocessed movie (3A) shows even EGFP recovery across the foci, whereas SIM- (3B) or *di*SIM- (3C) processed movies show uneven fluorescence recovery across the foci, revealing multiple subcompartments. The movie corresponds to cell shown in Figure 1. Scale bar as shown.

Next, we photobleached 53BP1-EGFP foci using a 473 nm laser to analyze the recovery of EGFP fluorescence. For this purpose, time-lapse images were acquired once every 5 s for 2 min using a leap mode, yielding 9 Z-slices. In *di*SIM-processed images, we observed a nonuniform recovery within the foci, leading to changes in the positions of EGFP intensity peaks, suggesting different protein diffusion rates within a single 53BP1 foci, indicating distinct subcompartments (Figure 1F). Thus, combining FRAP and SR imaging has the potential to reveal subcompartments of differing protein mobilities within larger subcellular structures.

### Few 53BP1 foci remain compact without separating into amorphous foci

**Figure 2:**
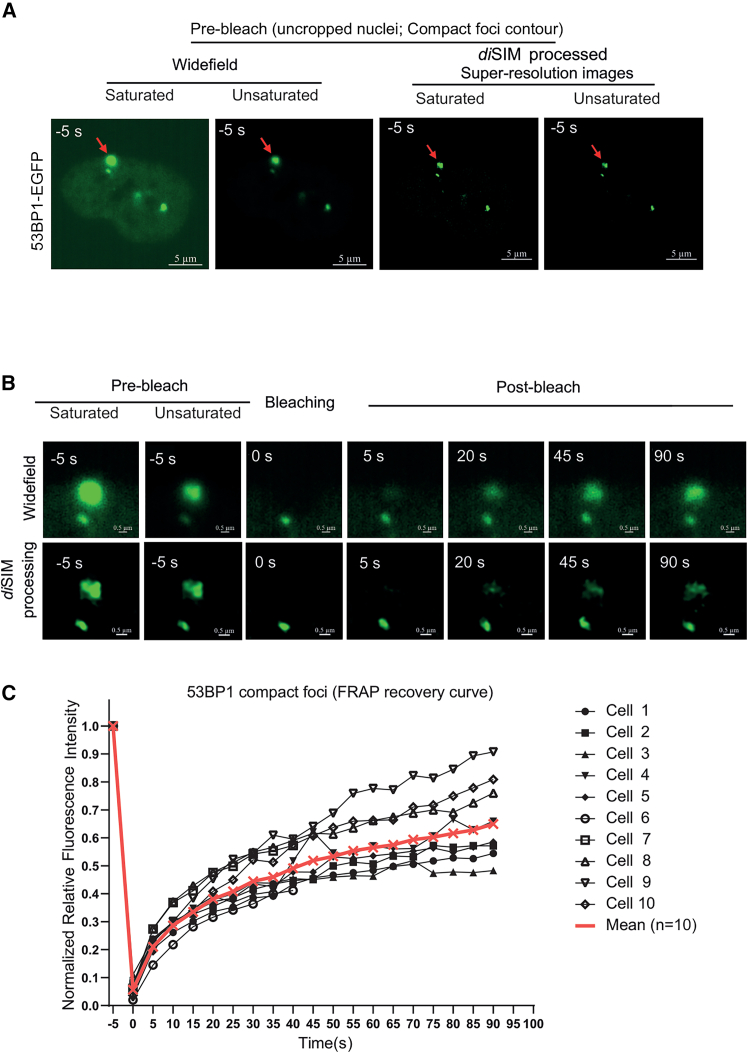
Single compact foci show a uniform recovery of 53BP1-EGFP signal (A) Representative wide-field and *di*SIM-processed pre-bleach images of nuclei with a compact foci (red arrow). Scale bars as indicated. (B) Cropped time-lapse images (wide-field or processed *di*SIM as indicated) show 53BP1 foci with a single compact contour photobleached to study FRAP rates. Scale bar as indicated. (C) Graph of relative fluorescence intensity of single-compartment 53BP1 foci shows FRAP of the first 100 s during the 3-min imaging period following photobleaching. Plateaus beyond 100 s are not shown. Saturation levels were set up for post-bleach recovery images (unsaturated pre-bleach images included). See also Video S4.

To assess differences in 53BP1 protein mobilities, we conducted FRAP measurements of 53BP1 foci in several cells and calculated FRAP rates using raw, unprocessed images. Nuclear foci that appeared as compact spots in pre-bleach images were first investigated (Figure 2A). *di*SIM-processed and unprocessed images of compact foci showed uniform recovery as a compact structure during the 2-min FRAP period, and these images were analyzed for 53BP1-EGFP recovery kinetics (Figure 2B; Videos S4A and S4B). In these compact 53BP1 foci, 53BP1-EGFP recovery kinetics (mean half-time of recovery [t_1/2_] = 19.67 s; *n* = 10 cells [Figure 2C]) are similar to what has been reported elsewhere.^10^ In compact foci, 55% of 53BP1-EGFP recovered within a minute, showing 53BP1 protein exchange (Figure 2C). These studies show that the FRAP rates of 53BP1-EGFP foci in our unprocessed SIM images are comparable to previous non-SIM studies. We conclude that 53BP1, within compact foci, exchanges uniformly as a single compartment.

Video S4. Series of unprocessed and diSIM-processed time-lapse movies of compact compartment 53BP1 foci in RPE1-TP53BP1-EGFP cells, related to Figure 2 A single 53BP1 foci (marked with red arrow) bleached at 5 s shows even fluorescence recovery across the foci, revealing a single compartment in both unprocessed (4A) and diSIM processed (4B) movies. The movie corresponds to the cell shown in Figure 2. Scale bar as shown.

### FRAP of 53BP1-EGFP indicates at least two types of 53BP1 foci

**Figure 3:**
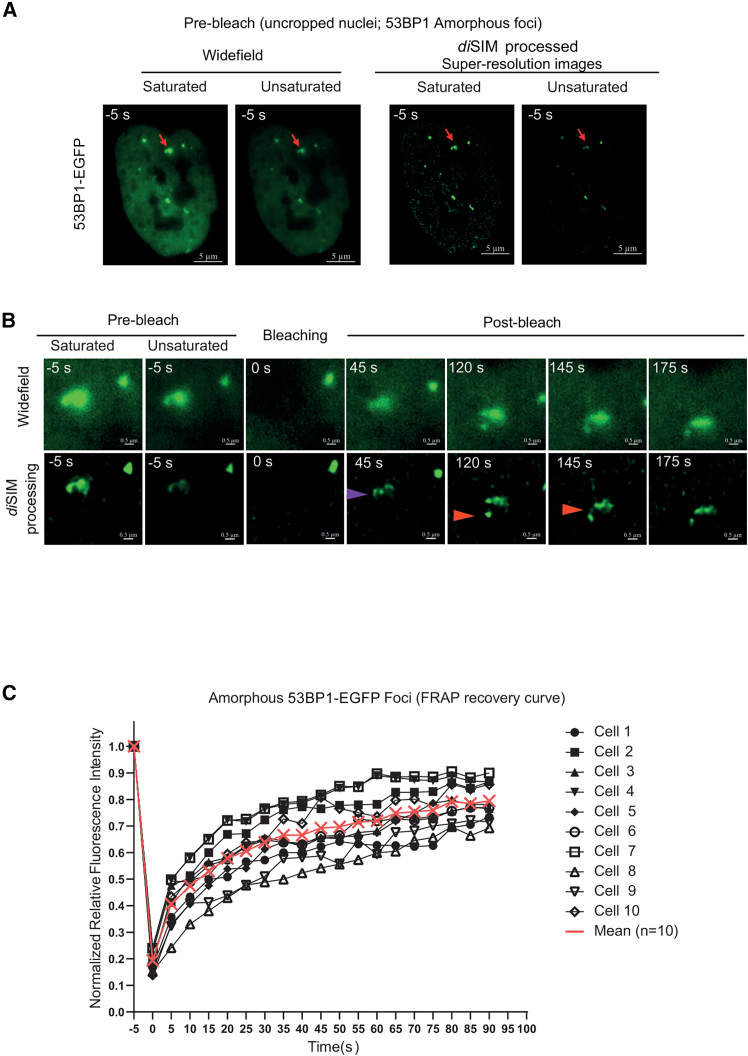
Amorphous 53BP1 foci show subcompartments and nonuniform recovery (A) Representative, unprocessed, or SIM- or *di*SIM-processed pre-bleach images of nuclei with amorphous foci (red arrow). Scale bar as indicated. (B) Cropped time-lapse images (raw or processed [SIM or *di*SIM as indicated]) show a multi-compartment 53BP1 foci that was photobleached to study FRAP rates. Purple arrowhead marks compartments that recover earlier, and red arrowhead tracks foci morphology and position change. Scale bar as indicated. (C) Graph of relative fluorescence intensity of multi-compartment 53BP1 foci shows FRAP during the 3-min imaging period following photobleaching. Saturation levels were set up for post-bleach recovery images (unsaturated pre-bleach images included). See also Video S5.

We next focused on 53BP1 foci that present amorphous and dynamically varying contours visible in both unprocessed and SIM-processed movies (Figure 3A; Video S5). We tested whether the FRAP rates of 53BP1 within the amorphous foci are similar to those in the compact foci. Following photobleaching, SIM-processed images showed differential recovery within the 53BP1 foci, with some regions (subcompartments) recovering faster than others (purple arrow, Figure 3B). The mean recovery kinetics of 53BP1-EGFP in the amorphous foci structures showed t_1/2_ = 15.58 s (*n* = 10 cells; Figure 3C). Occasionally, cells displayed both types of 53BP1 foci (compact and amorphous) within the same nuclei (Figure S1D). There was no significant increase in 53BP1 foci numbers during imaging (Figures S2A and S2B), suggesting that no significant DNA damage was introduced during the FRAP-SR imaging session.

Video S5. Series of unprocessed and diSIM-processed FRAP-SR of amorphous 53BP1 foci, related to Figure 3 Unprocessed and *di*SIM-processed images of 53BP1-EGFP foci (marked with red arrow) photobleached at 5 s show uneven fluorescence recovery across the foci, revealing multiple separable compartments. The movie corresponds to the cell shown in Figure 3. Scale bar as shown.

In summary, we conclude that 53BP1 foci can be separated into at least two different types based on (1) the amorphous or compact foci contour and (2) the presence of subcompartments with differing 53BP1 protein mobilities.

### 53BP1 foci displaying subcompartments may be more active than others

**Figure 4:**
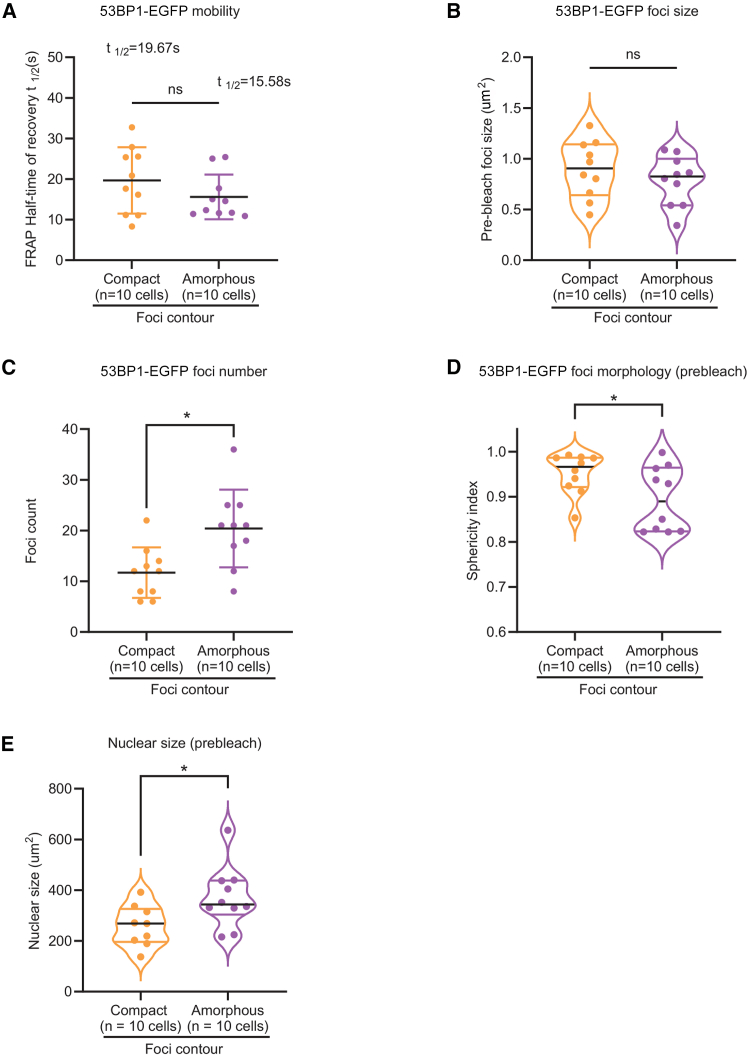
53BP1 foci contours, compact and amorphous, show an association with nuclear, but not foci, sizes (A) Graph showing the distribution of half-times of 53BP1-EGFP FRAP indicates an increased heterogeneity in recovery rates of single compact foci compared to amorphous foci. Two-tailed Mann-Whitney test, *p* = 0.3527, no significant difference. (B) Graph showing no significant difference in foci size (measured as area) between compact foci and amorphous foci (pre-bleach) in *di*SIM-processed images of RPE1 53BP1-EGFP cells (two-tailed Mann-Whitney test, *p* = 0.4359, no significant difference). (C) Graph showing some difference in 53BP1 foci numbers (measured as counts in pre-bleach nuclei) of SIM-processed images of cells displaying either compact foci or amorphous foci (two-tailed Mann-Whitney test, *p* = 0.1803, no significant difference). (D) Graph showing the sphericity index of 53BP1 foci (morphology of pre-bleach foci was considered for this analysis (unpaired t test, *p* = 0.0459, no significant difference). (E) Graph showing the distribution of nuclear sizes in cells corresponding to 53BP1 foci studied for FRAP rates in (A) (two-tailed Mann-Whitney test, *p* = 0.0279, significant difference; 1 cell with an unusually large nucleus was removed from the compact group). Median values are marked using black lines, and quartiles are marked using color lines.

FRAP rates of 53BP1-EGFP are more heterogeneous in compact foci than amorphous foci, suggesting varying protein mobilities,^28^ although there was no statistical significance between the two groups (Figure 4A). We set out to test if any other subcellular differences can be correlated to explain the subcompartments we observe in amorphous but not compact 53BP1 foci.

Large nuclear bodies with Oct1/PSE-binding transcription factor and transcription factors (OPT domains) 1.3 μm in diameter, appear in the G1 phase and disappear in the S phase.^15^^,^^29^ So first, we analyzed the sizes of the foci we photobleached. Both compact and amorphous foci exhibit a wide range of sizes from 0.34 to 1.33 μm^2^ as measured using *di*SIM-processed images. We find no significant difference in the sizes of the compact and amorphous foci (Figure 4B), showing that their activities may not strictly depend on the size of phase-separated structures.

53BP1-associated chromatin and DSB mobility have been reported.^30^^,^^31^^,^^32^ To test the extent of mobility in compact and amorphous foci, we measured foci displacement using centroids of the 53BP1 foci before and after photobleaching (to assess movement within 40 s) (Figure S3A). Five of the ten compact foci showed no displacement (Figures S3B and S3C; *n* = 20 cells). Although we did not find any statistical difference between the displacement of amorphous and compact foci, 50% of compact foci did not display any mobility, distinguishing the mobility likelihood of compact and amorphous foci.

Soon after mitosis, very few 53BP1 foci (as G1 bodies) are expected to arise from the previous cell cycle, whereas in the S phase, many more 53BP1 foci are expected.^6^^,^^14^^,^^33^ Therefore, we explored whether the number of foci within the nucleus differs in the cells that display compact or amorphous foci. Segmentation of images to automatically count 53BP1 foci based on EGFP intensities showed a moderate reduction in the total number of 53BP1 foci in cells with compact foci compared to those with amorphous foci (*n* = 20 cells) (Figure 4C). Next, we plotted the sphericity index to characterize the spikiness property of the foci’s exterior boundary. We found that compact foci tended to be more spherical compared to amorphous foci (Figure 4D). Lastly, we correlated the size of nuclei associated with amorphous or compact foci in an unbiased manner and showed a median 1.1-fold reduction in the nuclear size of cells displaying compact foci compared to amorphous foci (Figure 4E), suggesting cell-cycle-associated changes. Together, these quantitative studies show clear differences between 53BP1 foci contour, displacement propensity, and numbers, but not foci sizes, suggesting a closer link between nuclear size and foci contours compared to foci size per se.

### Lattice light-sheet movies show amorphous foci amid multiple 53BP1 foci induced by aphidicolin treatment

**Figure 5:**
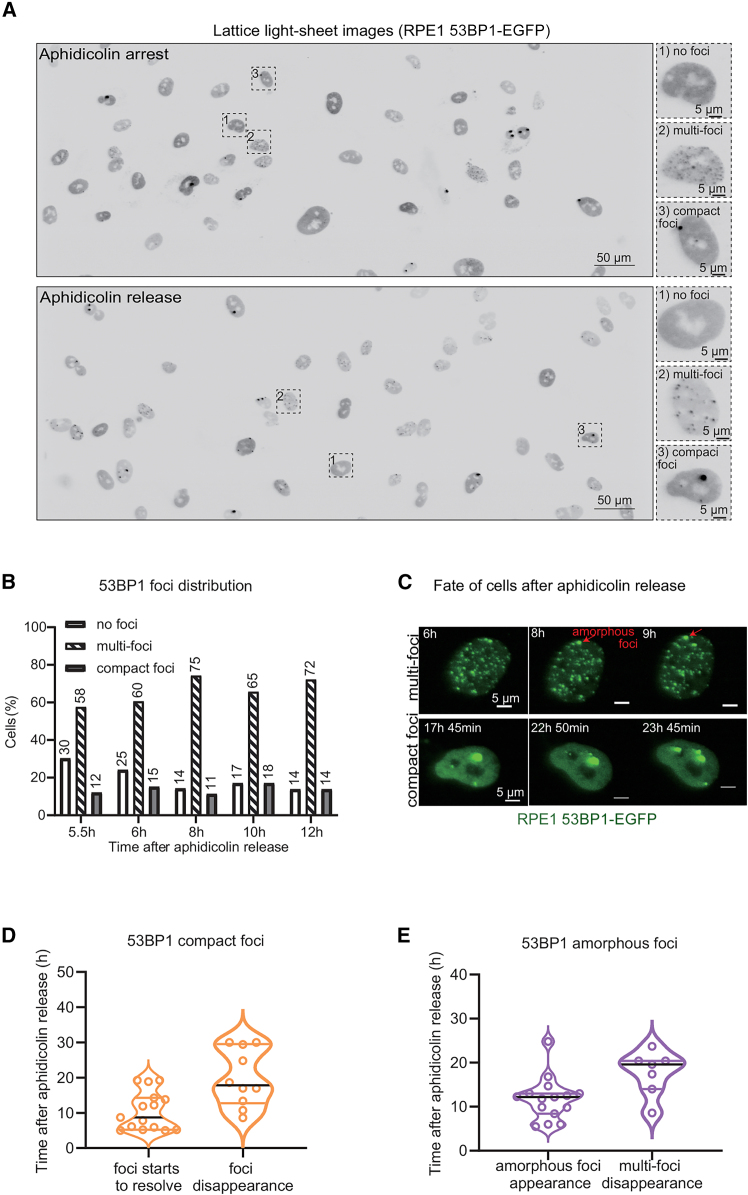
Lattice light-sheet imaging shows faster resolution of amorphous versus compact 53BP1 foci (A) Lattice light-sheet (LLS) images of cells released from aphidicolin treatment or retained in aphidicolin during imaging. Nuclei displaying no 53BP1 foci, compact foci, or amorphous foci are shown in magnified crops. Scale bar as indicated. (B) Bar graph showing the percentage of cells displaying no 53BP1 foci (no foci), multiple foci throughout the nuclei (multi-foci), or few compact foci. Numerical values on top of bars indicate percentage values (*n* = 33–36 cells/time frame). (C) Images show changes in the fate of foci in nuclei displaying multi-foci or compact foci. Amorphous foci showing dynamic changes in shape are marked in red. (D) Violin plot showing the beginning and end of compact foci resolution times following a release from aphidicolin treatment in cells expressing 53BP1-EGFP imaged using LLS microscopy as in (A). (E) Violin plot showing the appearance of amorphous foci and disappearance of multi-foci state after aphidicolin treatment release in movies of RPE1 cells expressing 53BP1-EGFP as in (A). Median values are marked using black lines, and quartiles are marked using colored lines.

Nuclear size changes through the cell cycle. To test whether differences in 53BP1 foci morphology are associated with cell cycle phases, we characterized 53BP1 foci occurrence and resolution times in long-term live-cell movies of aphidicolin-treated and -released cells. Using a lattice light-sheet microscope that offers a gentle illumination profile, we imaged RPE1 53BP1-EGFP once every 5 min for up to 24 h. Following aphidicolin treatment, we observed three types of nuclei based on the 53BP1 foci pattern: (1) no prominent foci (termed “no foci”), (2) one or two large compact foci, or (3) several diffused and small foci (termed multi-foci) (Figure 5A). 330 min after aphidicolin release, 30% of nuclei displayed no foci, 58% of nuclei displayed multi-foci, and 12% of nuclei displayed compact foci (Figure 5B). Dynamic changes in the number and size of compact foci were observed over time (Figure 5C). Amorphous foci were observed in nuclei displaying a multi-foci pattern (Figure 5C). Quantifying compact foci resolution times showed that the foci started to resolve as early as 5 h after aphidicolin release but showed a wide range of foci resolution times (Figure 5D). Similarly, G1 bodies that occur soon after mitosis and appear compact showed a range of foci resolution times (Figure S4). In multi-foci nuclei, we observed the appearance of amorphous 53BP1 foci but could not track their disappearance due to resolution limitations in lattice light-sheet microscopy (Figure 5E). However, we find that the multi-foci state of nuclei disappeared completely with time, suggesting cell cycle regulation (Figure 5E). In summary, the timing of foci appearance and foci resolution in cells released from aphidicolin suggests that 53BP1 foci morphology may reflect different protein activities or functional states of the foci through the cell cycle.

### FRAP-SR of amorphous foci in aphidicolin-released cells displays subcompartments of rapid 53BP1 recovery

**Figure 6:**
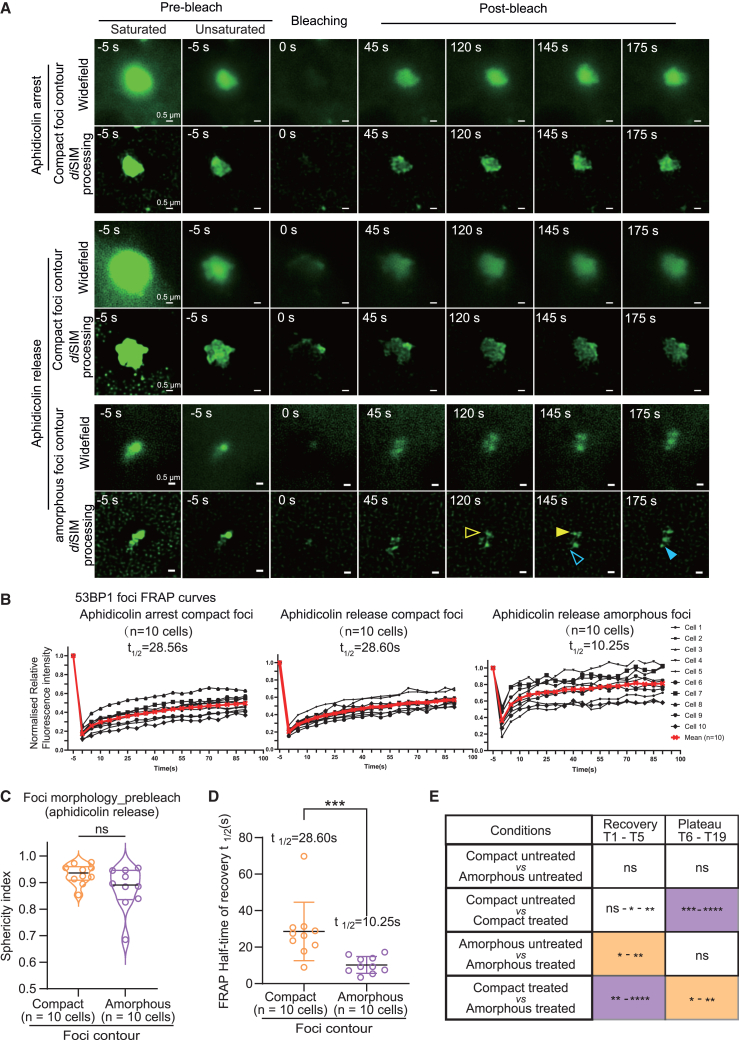
Rapid 53BP1 protein recovery in amorphous compared to compact foci following aphidicolin treatment (A) Cropped time-lapse images of compact or amorphous foci as indicated in aphidicolin-arrested or released cells. Saturation levels were set up for post-bleach recovery images (unsaturated pre-bleach images included). Post-recovery images (120–175 s) of amorphous 53BP1 foci continue to show uneven EGFP recovery (empty and filled color arrows mark the absence and presence of signal intensities, respectively). Scale bar as indicated. (B) Graphs of relative fluorescence intensity of 53BP1 foci show FRAP during the 3-min imaging period following photobleaching in compact or amorphous foci in aphidicolin-arrested or -released cells as indicated. t_1/2_ indicates half-maximal recovery times for each condition. (C) Violin plot showing sphericity indices of compact and amorphous 53BP1 foci, under pre-bleach conditions, in RPE1 cells released from aphidicolin treatment (two-tailed Mann-Whitney test, *p* = 0.0524, no significant difference). (D) Graph showing the distribution of half-times of 53BP1-EGFP FRAP indicating a faster recovery rate in amorphous compared to compact foci (two-tailed Mann-Whitney test, *p* = 0.0003, significant difference). (E) Table shows statistical differences extracted by comparing FRAP curves in two conditions indicated during signal recovery or plateau period as indicated in Figure S5C. Amorphous and compact foci in aphidicolin-treated and untreated conditions are compared. Two-way ANOVA was used to identify nonsignificant (ns) or significant differences among groups (∗∗∗∗∗*p* ≤ 0.00001, ∗∗∗∗*p* ≤ 0.0001, ∗∗∗*p* ≤ 0.001, ∗*p* ≤ 0.05, and ns *p* > 0.05). Median values are marked using black lines, and quartiles are marked using color lines.

Using FRAP-SR, we compared 53BP1-EGFP recovery in compact and amorphous foci of aphidicolin-arrested or -released cells (Figures 6A and S5A). In aphidicolin-arrested cells, we measured 53BP1 recovery only in compact foci (amorphous foci were not studied due to the crowding of multiple foci). In aphidicolin-release conditions (4–8 h after release), we measured 53BP1 recovery in compact and amorphous foci (Figure S5B). In the aphidicolin-release state, 53BP1-EGFP in amorphous foci recovered with a mean half-life of 10.25 s (Figure 6B). In aphidicolin-arrested or -release states, compact foci displayed a 53BP1 half-life of nearly 28.6 s (Figure 6B), suggesting slower 53BP1 protein exchange than compact foci in untreated cells (Figure 4A). Faster 53BP1 recovery rates in amorphous compared to compact foci following aphidicolin release (Figure 6B) indicate accelerated 53BP1 exchange in the subcompartments. These results were also reflected in immobile factions across conditions (Figure S5C). Although normalization of FRAP curves to assess the immobile fraction was difficult due to varying intensities of the foci, comparing normalized FRAP mean values across 10 cells showed that the immobile fraction in aphidicolin-arrested compact foci is the largest compared to untreated amorphous foci, which displayed the smallest immobile fraction across the tested conditions (Figure S5C). We observed no significant differences between foci morphologies, measured as the sphericity index (Figure 6C). However, the FRAP half-time recovery rates are significantly different between compact and amorphous foci in aphidicolin-release conditions (Figure 6D). We next compared the differences in FRAP rates of 53BP1-EGFP foci during the EGFP signal recovery period (T1–T5 time frames, less than 25 s after photobleaching) and the signal plateau period (T6–T19 time frames, more than 25 s after photobleaching) (Figure S5C). In alignment with half-time measurements (Figures 6D and 4A), compact and amorphous foci in aphidicolin-treated but not untreated cells responded significantly differently during the signal recovery period (Figure 6E). In contrast, compact foci in aphidicolin-treated versus untreated cells showed a significant difference in the recovery period (Figure 6E). These findings indicate differences in 53BP1 exchange, foci contours, and subcompartments in aphidicolin-treated and untreated cells, which may reflect differences in 53BP1 protein activities.

Combining FRAP and SR with additional markers for DNA damage repair would allow a deeper exploration of how DDR machinery access and occupancy are regulated. Collectively, the quantifications show the strength of FRAP-SR microscopy in comparing differences between 53BP1 foci contours, protein diffusion rates within subcompartments, and foci mobility for rigorously exploring protein activities and function within subcellular structures.

## Discussion

**Figure 7:**
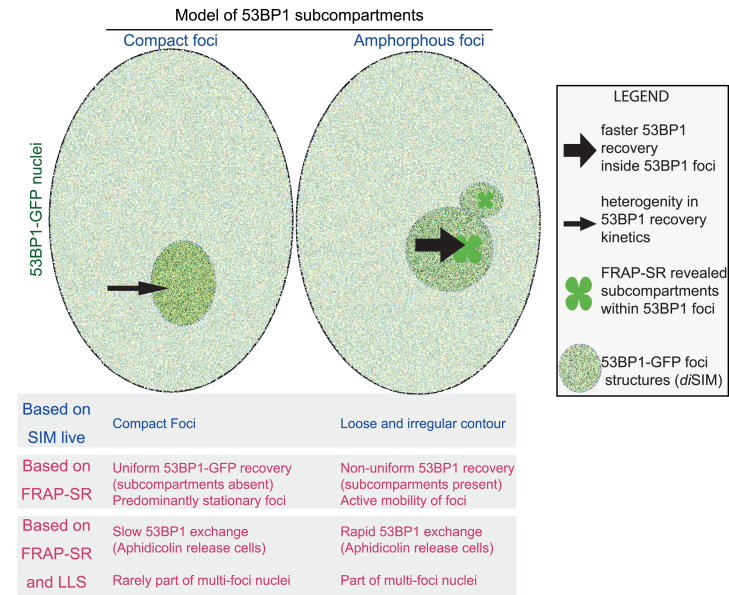
Model of subcompartments within 53BP1 foci showing sites of distinct activities based on differing 53BP1 protein diffusion rates FRAP in the SR regime shows (1) a slower 53BP1 exchange rate between foci and nucleoplasm compared to exchange within the foci and subcompartments and (2) heterogeneity in the recovery of EGFP-53BP1 signals following photobleaching of compact compared to amorphous foci. Differing protein mobilities and super-resolved structural dynamics may reflect distinct roles of 53BP1 activities and functions, as they correlate with different foci resolution kinetics. FRAP-SR can extend the imaging toolset to probe the dynamic regulation of subcellular structures.

We combine FRAP and SR microscopy studies to investigate 53BP1 protein exchange rates and subcellular structural changes in the super-resolution regime, taking a step beyond SR or FRAP studies done separately. Our SR live-cell studies of 53BP1 foci in unperturbed cells indicate that the foci can adopt either a compact or amorphous morphology. Amorphous foci show dynamic irregular shapes and increased foci movement, suggesting activity. FRAP studies demonstrate that protein mobilities within the amorphous foci can be uneven, leading to partial recovery of 53BP1 foci compartments and revealing subcompartments of differential protein activity (Figure 7). In contrast, 53BP1-EGFP in compact foci recovers uniformly. Following a release from DNA replication, 53BP1-EGFP recovers faster in amorphous than compact foci, indicating differences in 53BP1 protein activities. In addition to showcasing the strengths of FRAP-SR, our findings have conceptual implications on whether other components of the DDR machinery are uniformly found within the 53BP1 foci and whether different 53BP1 mobilities indicate differential roles in distinct subcellular events through the cell cycle (e.g., DNA replication or repair).

53BP1 foci can arise from different types of replication stress, clustered DNA damage, or non-DNA damage sites. Single-molecule localisation microscopy (SMLM) studies have shown the existence of subfoci of 53BP1, which arise from clustered DNA damage.^34^ Although 53BP1 has been shown to undergo phase separation,^10^^,^^11^ there was no prior knowledge of distinct protein mobilities within 53BP1 subcompartments, which we report here. Long-term imaging of cells after a FRAP-SR cycle could help measure and model foci and protein mobilities to explore whether compact foci follow sub-diffusive dynamics. Our FRAP-SR studies suggest that within phase-separated 53BP1 foci, there can be activity centers with increased protein mobility, which appear as subcompartments. Consistent with this model, in cells released from aphidicolin treatment, amorphous foci show faster 53BP1 protein exchange (FRAP-SR) compared to compact foci (Figure 6).

FRAP-SR studies using *di*SIM/SIM^2^ are strategically placed to resolve and link dynamic protein interactions and subcellular structural changes within a range of 60–120 nm with reduced phototoxicity (here and Efstathiou et al.^35^). High-throughput microscopy studies comparing the localization of isoforms, variants, and mutants of proteins across the cell cycle, especially mitosis, can benefit from a single-step FRAP-SR framework to explore protein mobilities/interactions in addition to dynamic changes in the subcellular structure they decorate.^36^^,^^37^^,^^38^^,^^39^ Structural changes due to frequent fusion and rare fission events have been reported to explain the droplet-like behavior of 53BP1 foci,^10^ wherein nuclear bodies and infra red (IR)-induced foci recover with similar kinetics, suggesting similar diffusion rates of 53BP1 molecules. In our study of aphidicolin-treated cells from different cell cycle stages, we find differences in the recovery kinetics of 53BP1 within compact and amorphous foci, indicative of distinct protein interactions and associated functions in these foci. Consistent with these findings, functional interrogation of DDR variants showed that 53BP1 mutants modulate protein binding without affecting the damage response.^40^

DNA break-repair studies have shown a fast component for resolving most of the breaks and a slow component for resolving some of the breaks.^6^^,^^41^^,^^42^^,^^43^ Whether the two different types of super-resolved 53BP1 foci indicate fast- and slow-resolving breaks (differing 53BP1 activities) can be confirmed with FRAP-SR of other DDR-associated proteins. Alternatively, a dynamic amorphous foci could indicate spaces of higher mobilities within the foci, consistent with BRCA1-mediated active exclusion of 53BP1 from DNA repair sites as proposed in previous SIM studies of BRCA1 and 53BP1.^17^^,^^19^ In our studies using unperturbed RPE1 cells, we ruled out foci arising from incomplete DNA replication or replication stress; these can be triggered by aphidicolin, which increases the incidence of 53BP1 foci in the G1 phase of the next cell cycle.^15^

High-speed nanoscale imaging of live cells has provided key insights (for example, organelle contact sites^44^ or induction of DSBs following radiation^31^^,^^45^). Also, high-speed volumetric imaging of live cells has enabled the probing of subcellular movements across mesoscales.^46^ Here, using lattice SIM, we combine volumetric SR methods and FRAP to reveal that not all 53BP1 foci display similar protein mobilities, and the foci that dynamically resolve into multiple compartments show increased 53BP1 protein mobility (Figure 7). Based on data from aphidicolin-treated and untreated cells, we propose that differing 53BP1 mobilities and foci structures may conceptually relate to different roles of 53BP1 within the foci as a mechanism well suited for varied spatiotemporal events. 53BP1 is involved in both rapid and slow cellular events: resolving DNA DSBs^45^^,^^47^ and fast chromatin mobilities^31^ are in the order of seconds and minutes, while 53BP1’s roles in mitosis and replication stress^48^ or heterochromatin binding^11^ are in the order of minutes to hours, requiring different types of interactors and regulatory mechanisms. Our foci displacement studies show that 75% of 53BP1 foci are mobile, allowing the foci to support long-range interactions. Long-range joining of DNA breaks, as in the distal joining of V(D)J mediated by 53BP1, is important, as defective cells can experience extensive degradation of the unrepaired coding ends,^13^ leading to genomic instability. In the long term, combining FRAP and *di*SIM can help exceed current limitations in studying photosensitive subcellular structures with varied protein mobilities, activities, and roles.

### Limitations of the study

The FRAP-SR method described here works well for subcellular structures that are larger than 60 nm. For those scenarios where structures are smaller than 60 nm, users may need to choose higher-resolution live-imaging methods that may present increased photoxicity. Second, FRAP-SR offers powerful insight into the compartmentalization of diffraction-limited structures. However, FRAP-SR may not be readily scalable to hundreds of precisely targeted imaging events, as required for modeling studies,^49^^,^^50^ in the absence of automated foci-targeting optoelectronic tools. Third, 53BP1 is an abundant protein that enables our analysis of EGFP foci signals, and if users are working with less-abundant proteins, they may need brighter fluorophores, such as mNeonGreen.

## Resource availability

### Lead contact

Requests for further information and resources should be directed to and will be fulfilled by the lead contact, Viji M. Draviam (v.draviam@qmul.ac.uk).

### Materials availability

Cell lines in the study are generated by Horizon/Revitty. They can be shared for academic collaboration with compensation for its processing and shipping. Reagents in this study can be accessed through the lead contact.

### Data and code availability

• Representative raw data have been deposited at Zenodo and are publicly available at https://zenodo.org/records/13941719 (DOI: https://doi.org/10.5281/zenodo.13941719). Data reported in this work will be shared by the lead contact upon request.
• This paper does not report original code.
• Any additional information required to reanalyze the data reported in this work is available from the lead contact upon request.

## Acknowledgments

We acknowledge funding support from BBSRC, UKRI (BBR01003X/1, BB/W002698/1, BB/V018310/1, and BBT017716/1 to V.M.D.); MRC, UKRI (MR/X013847/1 to V.M.D.); QMUL (SBC8DRA2 and SBC9DRA2 to V.M.D.); a QMUL-ZEISS joint PhD studentship to M.C; a CSC scholarship to S.D. (no. 202206320050); a CR UK fellowship (C28598/A9787) to V.M.D.; and a CONACYT scholarship to J.C.M.-G. (CVU no. 1042679). We acknowledge Sam Court and Petra Ungerer for infrastructure maintenance support. We thank Christoforos Efstathiou, Bint-E-Zainab, and Vidula Vallari Sastry for their comments and constructive feedback.

## Author contributions

V.M.D. designed the study and drafted the manuscript. C.W. helped edit the manuscript with V.M.D. C.W. conducted all the FRAP, SIM, and LLS7 imaging studies. C.W. analyzed the images and generated the figures for all panels unless indicated otherwise. J.C.M.-G. set up the ArivisPro foci segmentation framework to analyze foci numbers (Figures 4C and S2B) and generated Figure 6E; S.D. analyzed G1 bodies and prepared Figure S4; C.W. and M.C. jointly analyzed Figures 2C, 3C, 4A, and 5B; N.S. conducted immunoblotting studies to characterize RPE1 cell lines for 53BP1-EGFP or Mad1-EGFP expression. V.M.N. standardized and performed aphidicolin treatment and washes for the aphidicolin release study (Figures 5 and 6). S.D. cultured RPE1 for FRAP studies in Figures 1, 2, 3, and 4. Statistical analyses were performed by J.C.M.-G. and C.W. All authors commented on the manuscript.

## Declaration of interests

The authors declare that there are no competing interests.

## STAR★Methods

### Key resources table

**Table:** 

REAGENT or RESOURCE	SOURCE	IDENTIFIER
**Chemicals, peptides, and recombinant proteins**		

aphidicolin	Merck	Cat#178273-1MG
Leibovitz’s L15 medium	Invitrogen	Cat# 11415064
60nm DNA origami, Alexa Fluor 488 Dye	GATTAquant GmbH	HS-Code: 3822.00.00

**Deposited data**		

Raw and analyzed data	This paper	https://zenodo.org/records/13941719

**Experimental models: Cell lines**		

CRISPR/Cas9 RPE1 53BP1-eGFP (clone 213) cells also called RPE1 53BP1-EGFP cells	Horizon Discovery Ltd	Cat# HD 614-016
RPE1 Mad1_L1-eGFP (clone 73) cells, also called RPE1 Mad1_L1-EGFP cells	Horizon Discovery Ltd	Cat# HD 614-007

**Oligonucleotides**		

Primers used in the study	This paper	See Table S1B

**Software and algorithms**		

ImageJ	Schneider et al.^51^	https://imagej.nih.gov/ij/
Arivis Vision4D 4.1.2	ZEISS	https://kb.arivis.com/arivis-vision4d-4.1.2-release-notes-november-16-2023
Adobe illustrator 2024	Adobe	https://www.adobe.com/uk/products/illustrator.html
ZEN blue 3.4	ZEISS	Carl Zeiss ZEN 3.4(blue edition)
ZEN black 3.0	ZEISS	Carl Zeiss ZEN 3.0SR FP2(black) Elyra Release Version 16.0.0.0
Graph Pad Prism 9™	Dotmatics	https://www.graphpad.com
BioRender	BioRender	https://www.biorender.com

### Experimental model and study participant details

#### Cell culture and media for CRISPR-Engineered RPE1 TP53BP1-EGFP clones

CRISPR/Cas9 engineered cell lines are purchased from Horizon/Revitty. Briefly, CRISPR/Cas9 was used to engineer the endogenous locus of either TP53BP1 or Mad1_L1 gene of hTERT-RPE1 cell line to integrate an in-frame sequence encoding eGFP. DNA primers used by Horizon/Revitty to confirm integration are presented in Table S1. RPE1 53BP1-eGFP (clone 213) cells and RPE1 Mad1_L1-eGFP (clone 73) cells, also called 53BP1-EGFP and Mad1_L1-EGFP cells, respectively, were cultured in plastic dishes (Corning 430641U) and grown in DMEM/F12 media (21331-046) in a 37° incubator. For aphidicolin treatment, the cells were treated with 1 microM aphidicolin overnight (10–16 h). The aphidicolin release group was washed 5 times with 15–20 min of incubation at 37° Celsius and then transferred to Leibovitz’s L15 medium for imaging. The aphidicolin arrest group was transferred to Leibovitz’s L15 medium with 1 microM aphidicolin.

### Method details

#### Lattice Lightsheet (LLS7) imaging

For live-cell imaging using LLS7 (Lattice Lightsheet 7, ZEISS), RPE1 cells were seeded onto 4-well cover glass chambered dishes (Lab-Tek; 1064716) or 4-well glass bottom ibidi dish, and transferred to Leibovitz’s L15 medium (Invitrogen: 11415064) for imaging. For live-cell studies, 20,000 cells were seeded in each well 24 h before imaging. Imaging was performed at 37°C using a full-stage incubation chamber setup to allow normal mitosis progression and cell cycle dynamics.

#### FRAP & diSIM/SIM2 (Elyra7- RappOpto) imaging

For super-resolution live-cell imaging and FRAP, using Elyra 7 (ZEISS, Jena, Germany) equipped with UGA-42 Firefly (Rapp OptoElectronic GmbH, Germany), cells were seeded onto a 4-well ibidi glass-bottom dish (ibidi; 80427) for 24 h and then changed to Leibovitz’s L15 medium (Invitrogen; 11415064) before imaging. Imaging was performed at 37°C using a full-stage incubation chamber, with a 5-s time interval, 3 Z planes, 0.5 μm apart, in leap mode (9 Z-slice SIM), and acquired using a 63×/1.4 oil immersion objective. For photobleaching studies, a 473 nm laser was used at 100% power for 0.5 s. The Elyra 7 features Lattice SIM (Structured Illumination Microscopy) equipped with a *di*SIM image reconstruction algorithm, allowing fast and gentle super-resolution imaging (resolution of ∼60 nm in xy and a leap mode of accelerated volume imaging). Images were acquired with a 16-bit 512 × 512 pixel PCO.edge 4.2 sCMOS. 60nm DNA origami (GATTAquant GmbH, Alexa Fluor 488 Dye) was used to assess the resolution of the setup.

### Quantification and statistical analysis

#### Particle size analysis and displacement

Automated particle analysis to count foci numbers in each cell was performed using Arivis software (Arivis Vision4D 4.1.2, ZEISS). For this, one representative SIM-processed image was selected, and a pipeline was generated by setting an intensity threshold for the segmentation of particles; Particles were classified based on mean intensity using an object feature filter. Once the pipeline was generated using one image, all *di*SIM/SIM^2^-processed movies were analyzed with the same pipeline.

For semi-automated measurements of the pre-bleached 53BP1 foci particle size, we used ZEN software for *di*SIM processing and z stack maximum projection, then used the Draw spline contour from the graphics tool to measure the area of the particles. The displacement of 53BP1 foci particles was measured as absolute displacement between the foci centroids at 5 s before bleaching and 40 s after recovery. The morphology measurements of 53BP1 foci were conducted with FIJI (ImageJ)^52^_ Analyze Particles tool to measure the perimeter and the Convex Hull of the foci. The ratio between the convex hull perimeter and ROI foci perimeter is a measure of the sphericity or ‘spikiness’. If the ratio is close to 1, then the foci are almost spherical; the closer the ratio goes toward zero, the spikier the foci.

Immunoblotting was performed on proteins separated on 8% SDS-PAGE gels by transferring them overnight onto PVDF membranes. Lysates were generated by treating cells with a Benzonase lysis buffer consisting of 75 mM HEPES, 150 mM NaCl, 1.5 mM EGTA, 10 mM MgCl_2_, 10% Glycerol, 0.1% NP-40 and Benzonase 90 U/ml. Membranes were incubated in primary antibodies against GFP (Abcam, ab290; 1:1000) and probed using secondary antibodies labeled with infrared fluorescent dyes, which were imaged using an iBright 1500 imager.

#### SIM and FRAP analysis

SIM acquisition and *di*SIM processing were performed using ZEN Black software. Images and Movies were prepared using ZEN Blue software. Additional analysis of FRAP intensity measurement was conducted on Fiji,^51^ Microsoft Excel, and graphs were plotted using GraphPad Prism 9. The equation for FRAP normalised relative fluorescence intensity calculation are.

(1) Step 1. Photobleaching Rate (r) = (Fc-Fb)/(Fc_0_-Fb)
(2) Step 2. Recovery Rate of ROI (R) = (Fi-Fb)/(Fi_0_-Fb)
(3) Step 3. Normalised Recovery Rate of ROI = R/r

Fi represents the fluorescence intensity region of interest, Fi_0_ represents the fluorescence intensity region of interest before bleaching, Fc represents the Fluorescence intensity control, Fc_0_ represents the Fluorescence intensity control before bleaching, Fb represents the Fluorescence intensity background, and ROI represents the region of interest.

Statistical analysis was performed in Graph Pad Prism 9 using statistics: Non-linear fit, and one-phase association analysis to determine half-life recovery of fluorescence intensity per compact and amorphous foci in FRAP. Independent group samples unpaired t-test and nonparametric tests, i.e., two-tailed Mann-Whitney test were used to determine the significance of any differences observed.

Two-way ANOVA was used to make comparisons among groups (∗∗∗*p* ≤ 0.001, ∗*p* ≤ 0.05, ns *p* > 0.05).

## Supplemental information

Document S1. Figures S1–S5 and Tables S1

Document S2. Article plus supplemental information

## References

[1] Draviam V.M., Xie S., Sorger P.K. (2004). Chromosome segregation and genomic stability. Curr. Opin. Genet. Dev..

[2] Huyen Y., Zgheib O., Ditullio R.A., Gorgoulis V.G., Zacharatos P., Petty T.J., Sheston E.A., Mellert H.S., Stavridi E.S., Halazonetis T.D. (2004). Methylated lysine 79 of histone H3 targets 53BP1 to DNA double-strand breaks. Nature.

[3] Zgheib O., Pataky K., Brugger J., Halazonetis T.D. (2009). An oligomerized 53BP1 tudor domain suffices for recognition of DNA double-strand breaks. Mol. Cell Biol..

[4] Wilson M.D., Benlekbir S., Fradet-Turcotte A., Sherker A., Julien J.-P., McEwan A., Noordermeer S.M., Sicheri F., Rubinstein J.L., Durocher D. (2016). The structural basis of modified nucleosome recognition by 53BP1. Nature.

[5] Fradet-Turcotte A., Canny M.D., Escribano-Díaz C., Orthwein A., Leung C.C.Y., Huang H., Landry M.-C., Kitevski-LeBlanc J., Noordermeer S.M., Sicheri F., Durocher D. (2013). 53BP1 is a reader of the DNA-damage-induced H2A Lys 15 ubiquitin mark. Nature.

[6] Schultz L.B., Chehab N.H., Malikzay A., Halazonetis T.D. (2000). p53 binding protein 1 (53BP1) is an early participant in the cellular response to DNA double-strand breaks. J. Cell Biol..

[7] Wang B., Matsuoka S., Carpenter P.B., Elledge S.J. (2002). 53BP1, a mediator of the DNA damage checkpoint. Science.

[8] Hart M., Adams S.D., Draviam V.M. (2021). Multinucleation associated DNA damage blocks proliferation in p53-compromised cells. Commun. Biol..

[9] Houtsmuller A.B., Rademakers S., Nigg A.L., Hoogstraten D., Hoeijmakers J.H., Vermeulen W. (1999). Action of DNA repair endonuclease ERCC1/XPF in living cells. Science.

[10] Kilic S., Lezaja A., Gatti M., Bianco E., Michelena J., Imhof R., Altmeyer M. (2019). Phase separation of 53BP1 determines liquid-like behavior of DNA repair compartments. EMBO J..

[11] Zhang L., Geng X., Wang F., Tang J., Ichida Y., Sharma A., Jin S., Chen M., Tang M., Pozo F.M. (2022). 53BP1 regulates heterochromatin through liquid phase separation. Nat. Commun..

[12] Bothmer A., Robbiani D.F., Feldhahn N., Gazumyan A., Nussenzweig A., Nussenzweig M.C. (2010). 53BP1 regulates DNA resection and the choice between classical and alternative end joining during class switch recombination. J. Exp. Med..

[13] Difilippantonio S., Gapud E., Wong N., Huang C.-Y., Mahowald G., Chen H.T., Kruhlak M.J., Callen E., Livak F., Nussenzweig M.C. (2008). 53BP1 facilitates long-range DNA end-joining during V(D)J recombination. Nature.

[14] Lukas C., Savic V., Bekker-Jensen S., Doil C., Neumann B., Pedersen R.S., Grøfte M., Chan K.L., Hickson I.D., Bartek J., Lukas J. (2011). 53BP1 nuclear bodies form around DNA lesions generated by mitotic transmission of chromosomes under replication stress. Nat. Cell Biol..

[15] Harrigan J.A., Belotserkovskaya R., Coates J., Dimitrova D.S., Polo S.E., Bradshaw C.R., Fraser P., Jackson S.P. (2011). Replication stress induces 53BP1-containing OPT domains in G1 cells. J. Cell Biol..

[16] Heemskerk T., van de Kamp G., Essers J., Kanaar R., Paul M.W. (2023). Multi-scale cellular imaging of DNA double strand break repair. DNA Repair.

[17] Chapman J.R., Sossick A.J., Boulton S.J., Jackson S.P. (2012). BRCA1-associated exclusion of 53BP1 from DNA damage sites underlies temporal control of DNA repair. J. Cell Sci..

[18] Depes D., Lee J.-H., Bobkova E., Jezkova L., Falkova I., Bestvater F., Pagacova E., Kopecna O., Zadneprianetc M., Bacikova A. (2018). Single-molecule localization microscopy as a promising tool for γH2AX/53BP1 foci exploration. Eur. Phys. J. D.

[19] Whelan D.R., Rothenberg E. (2021). Super-resolution mapping of cellular double-strand break resection complexes during homologous recombination. Proc. Natl. Acad. Sci. USA.

[20] Mudumbi K.C., Czapiewski R., Ruba A., Junod S.L., Li Y., Luo W., Ngo C., Ospina V., Schirmer E.C., Yang W. (2020). Nucleoplasmic signals promote directed transmembrane protein import simultaneously via multiple channels of nuclear pores. Nat. Commun..

[21] Mudumbi K.C., Schirmer E.C., Yang W. (2016). Single-point single-molecule FRAP distinguishes inner and outer nuclear membrane protein distribution. Nat. Commun..

[22] Löschberger A., Novikau Y., Netz R., Spindler M.-C., Benavente R., Klein T., Sauer M., Kleppe I. (2021). Super-Resolution Imaging by Dual Iterative Structured Illumination Microscopy. bioRxiv.

[23] Guo Y., Li D., Zhang S., Yang Y., Liu J.-J., Wang X., Liu C., Milkie D.E., Moore R.P., Tulu U.S. (2018). Visualizing Intracellular Organelle and Cytoskeletal Interactions at Nanoscale Resolution on Millisecond Timescales. Cell.

[24] Mythily D.V., Krishna S., Tergaonkar V. (1999). Pleiotropic effects of human papillomavirus type 16 E6 oncogene expression in human epithelial cell lines. J. Gen. Virol..

[25] Tergaonkar V., Mythily D.V., Krishna S. (1997). Cytokeratin patterns of expression in human epithelial cell lines correlate with transcriptional activity of the human papillomavirus type 16 upstream regulatory region. J. Gen. Virol..

[26] Chen B.-C., Legant W.R., Wang K., Shao L., Milkie D.E., Davidson M.W., Janetopoulos C., Wu X.S., Hammer J.A., Liu Z. (2014). Lattice light-sheet microscopy: imaging molecules to embryos at high spatiotemporal resolution. Science.

[27] Jullien D., Vagnarelli P., Earnshaw W.C., Adachi Y. (2002). Kinetochore localisation of the DNA damage response component 53BP1 during mitosis. J. Cell Sci..

[28] Lippincott-Schwartz J., Snapp E.L., Phair R.D. (2018). The development and enhancement of FRAP as a key tool for investigating protein dynamics. Biophys. J..

[29] Pombo A., Cuello P., Schul W., Yoon J.B., Roeder R.G., Cook P.R., Murphy S. (1998). Regional and temporal specialization in the nucleus: a transcriptionally-active nuclear domain rich in PTF, Oct1 and PIKA antigens associates with specific chromosomes early in the cell cycle. EMBO J..

[30] Lottersberger F., Karssemeijer R.A., Dimitrova N., de Lange T. (2015). 53BP1 and the LINC Complex Promote Microtubule-Dependent DSB Mobility and DNA Repair. Cell.

[31] Faustini E., Panza A., Longaretti M., Lottersberger F. (2024). Quantitative analysis of nuclear deformations and DNA damage foci dynamics by live-cell imaging. Methods Cell Biol..

[32] Dimitrova N., Chen Y.-C.M., Spector D.L., de Lange T. (2008). 53BP1 promotes non-homologous end joining of telomeres by increasing chromatin mobility. Nature.

[33] Lezaja A., Panagopoulos A., Wen Y., Carvalho E., Imhof R., Altmeyer M. (2021). RPA shields inherited DNA lesions for post-mitotic DNA synthesis. Nat. Commun..

[34] Bobkova E., Depes D., Lee J.-H., Jezkova L., Falkova I., Pagacova E., Kopecna O., Zadneprianetc M., Bacikova A., Kulikova E. (2018). Recruitment of 53BP1 Proteins for DNA Repair and Persistence of Repair Clusters Differ for Cell Types as Detected by Single Molecule Localization Microscopy. Int. J. Mol. Sci..

[35] Efstathiou C., Ojkic N., Draviam V.M. (2025). Dynein synergises with EB1 to facilitate cortex-microtubule encounter and proper spindle positioning in metaphase. bioRxiv.

[36] Islam A., Manjarrez-González J.C., Song X., Gore T., Draviam V.M. (2024). Search for chromosomal instability aiding variants reveal naturally occurring kinetochore gene variants that perturb chromosome segregation. iScience.

[37] Zulkipli I., Clark J., Hart M., Shrestha R.L., Gul P., Dang D., Kasichiwin T., Kujawiak I., Sastry N., Draviam V.M. (2018). Spindle rotation in human cells is reliant on a MARK2-mediated equatorial spindle-centering mechanism. J. Cell Biol..

[38] Song X., Conti D., Shrestha R.L., Braun D., Draviam V.M. (2021). Counteraction between Astrin-PP1 and Cyclin-B-CDK1 pathways protects chromosome-microtubule attachments independent of biorientation. Nat. Commun..

[39] Hart M., Zulkipli I., Shrestha R.L., Dang D., Conti D., Gul P., Kujawiak I., Draviam V.M. (2019). MARK2/Par1b kinase present at centrosomes and retraction fibres corrects spindle off-centring induced by actin disassembly. Open Biol..

[40] Cuella-Martin R., Hayward S.B., Fan X., Chen X., Huang J.-W., Taglialatela A., Leuzzi G., Zhao J., Rabadan R., Lu C. (2021). Functional interrogation of DNA damage response variants with base editing screens. Cell.

[41] Löbrich M., Rydberg B., Cooper P.K. (1995). Repair of x-ray-induced DNA double-strand breaks in specific Not I restriction fragments in human fibroblasts: joining of correct and incorrect ends. Proc. Natl. Acad. Sci. USA.

[42] Núñez M.I., Villalobos M., Olea N., Valenzuela M.T., Pedraza V., McMillan T.J., Ruiz de Almodóvar J.M. (1995). Radiation-induced DNA double-strand break rejoining in human tumour cells. Br. J. Cancer.

[43] DiBiase S.J., Zeng Z.C., Chen R., Hyslop T., Curran W.J., Iliakis G. (2000). DNA-dependent protein kinase stimulates an independently active, nonhomologous, end-joining apparatus. Cancer Res..

[44] Obara C.J., Nixon-Abell J., Moore A.S., Riccio F., Hoffman D.P., Shtengel G., Xu C.S., Schaefer K., Pasolli H.A., Masson J.-B. (2024). Motion of VAPB molecules reveals ER–mitochondria contact site subdomains. Nature.

[45] Sisario D., Memmel S., Doose S., Neubauer J., Zimmermann H., Flentje M., Djuzenova C.S., Sauer M., Sukhorukov V.L. (2018). Nanostructure of DNA repair foci revealed by superresolution microscopy. FASEB J..

[46] Efstathiou C., Draviam V.M. (2021). Electrically tunable lenses - eliminating mechanical axial movements during high-speed 3D live imaging. J. Cell Sci..

[47] Lou J., Priest D.G., Solano A., Kerjouan A., Hinde E. (2020). Spatiotemporal dynamics of 53BP1 dimer recruitment to a DNA double strand break. Nat. Commun..

[48] Bleiler M., Cyr A., Wright D.L., Giardina C. (2023). Incorporation of 53BP1 into phase-separated bodies in cancer cells during aberrant mitosis. J. Cell Sci..

[49] Corrigan A.M., Shrestha R., Draviam V.M., Donald A.M. (2015). Modeling of noisy spindle dynamics reveals separable contributions to achieving correct orientation. Biophys. J..

[50] Dang D., Efstathiou C., Sun D., Yue H., Sastry N.R., Draviam V.M. (2023). Deep learning techniques and mathematical modeling allow 3D analysis of mitotic spindle dynamics. J. Cell Biol..

[51] Schindelin J., Arganda-Carreras I., Frise E., Kaynig V., Longair M., Pietzsch T., Preibisch S., Rueden C., Saalfeld S., Schmid B. (2012). Fiji: an open-source platform for biological-image analysis. Nat. Methods.

[52] Schneider C.A., Rasband W.S., Eliceiri K.W. (2012). NIH Image to ImageJ: 25 years of image analysis. Nat. Methods.

