# Supplementary material for: Fluorescence recovery in the super-resolution regime reveals subcompartments of 53BP1 foci
Source: Cell Rep Methods. 2025 Aug 4;5(8):101118. doi: 10.1016/j.crmeth.2025.101118 (PMC12461648; doi:10.1016/j.crmeth.2025.101118)
Supplement: Document S1. Figures S1–S5 and Tables S1 [file mmc1.pdf]

**Cell Reports Methods, Volume *5***

## Supplemental information

# Fluorescence recovery in the super-resolution regime reveals subcompartments of 53BP1 foci

**Chengchen Wu, Janeth Catalina Manjarrez-González, Muntaqa Choudhury, Noor Shamkhi, Siwen Ding, Vishnu M. Nair, and Viji M. Draviam**

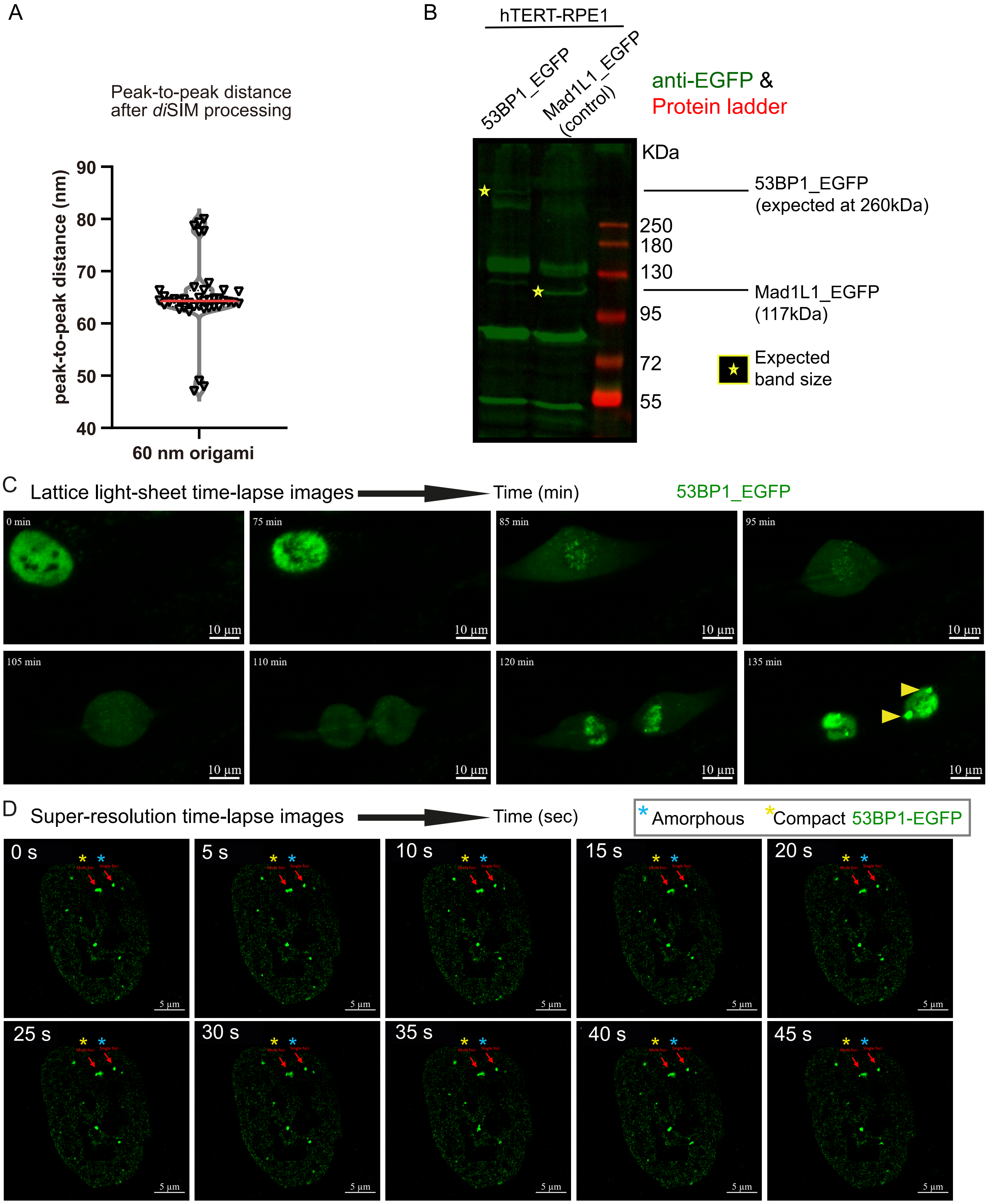


**Figure S1: Characterisation of *di*SIM lateral resolution and CRISPR-engineered hTERT-RPE1 53BP1-EGFP cell line, related to Figure 1.**

**A)** Distribution of distance between the twin foci observed following diSIM processing of 60 nm origami beads (data relates to images in Figure 1A). **B)** Immunoblot of lysates of RPE1 cells CRISPR engineered either at the TP53BP1 or MAD1L1 gene locus to introduce a C-terminal EGFP tag. Immunoblots probed with anti-EGFP antibodies (pseudo-coloured in green) show the expression of 53BP1-EGFP or Mad1L1-EGFP as expected. In red are protein marker lanes with estimated molecular weights marked on the right. Mad1L1-EGFP lysate is used as a control. The immunoblot is related to the grayscale image presented in Figure 1D. **C)** Time-lapse LLS7 images show 53BP1-EGFP foci appearing normally as G1 bodies soon after mitosis. Images of a G2 phase interphase cell entering mitosis and disassembling 53BP1 foci (Figure related to Movie S1). Yellow arrowheads mark G1 bodies. **D)** Super-resolution *di*SIM processed time-lapse images of an RPE1 53BP1-EGFP interphase nuclei show two types of 53BP1-EGFP foci: right foci remains as compact foci (blue asterisk) while on the left, an amorphous foci (yellow asterisk) shows a dynamic irregular foci contour growing and shrinking (red arrows mark compact or amorphous foci). No photobleaching was conducted. Scale bars as shown.

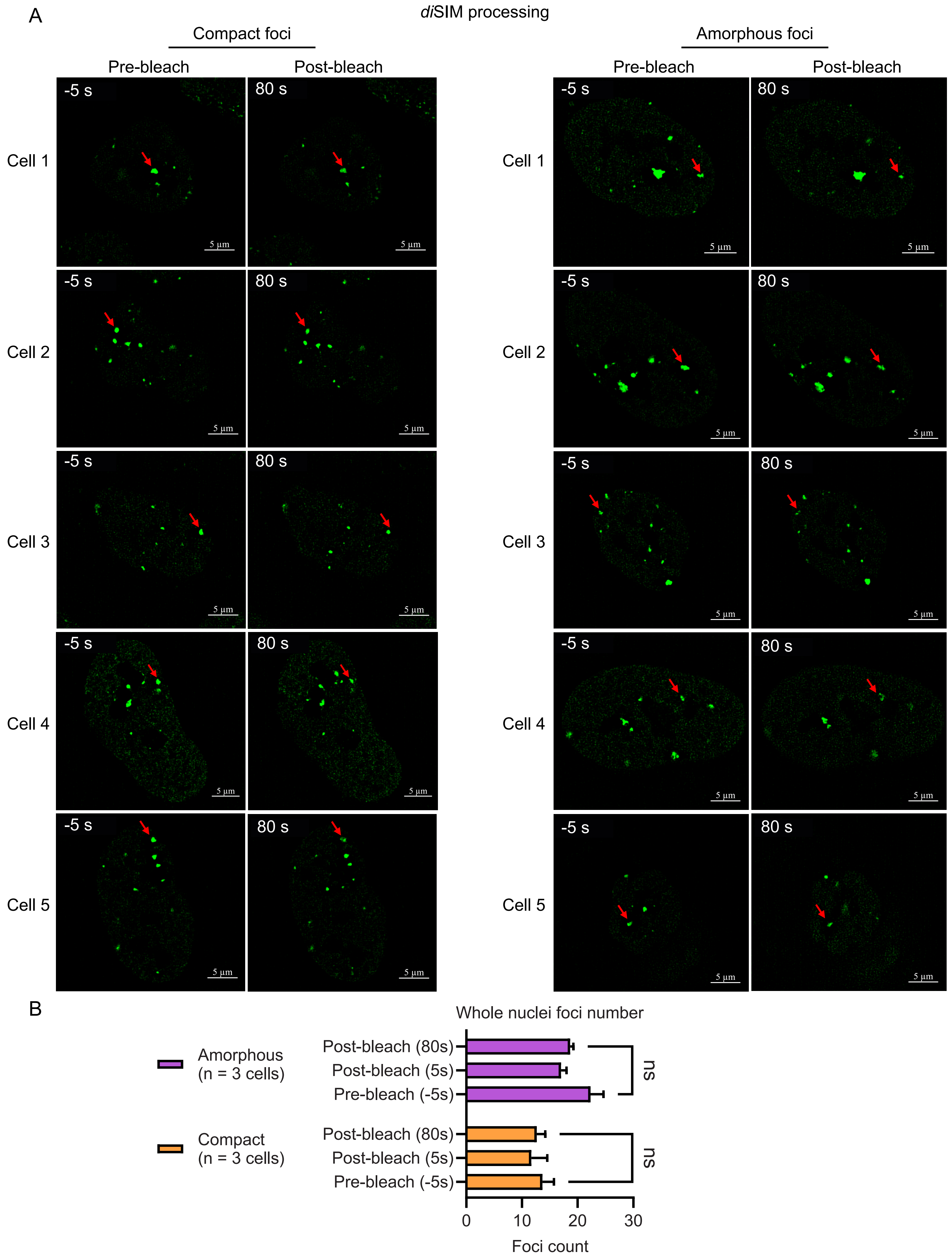


**Figure S2: No increase in foci count during FRAP-SR studies, related to Figures 2 and 3.**

**A)** Super-resolution *di*SIM images of compact and amorphous foci. Five representative images from the FRAP study (pre- and post-bleaching) are presented to showcase a) full recovery of 53BP1-EGFP b) uniform recovery on the left (compact foci) c) multi-compartments on the right (amorphous). Scale bars as shown. **B)** 53BP1 foci count, in compact or amorphous foci-bearing nuclei, measured using SIM-processed 53BP1-EGFP images showing no significant increase in foci count following bleaching. Pre-bleach and Post-bleach (5 or 80s after bleaching) foci count shown. Nonsignificant 'ns' differences were estimated using two way ANOVA.

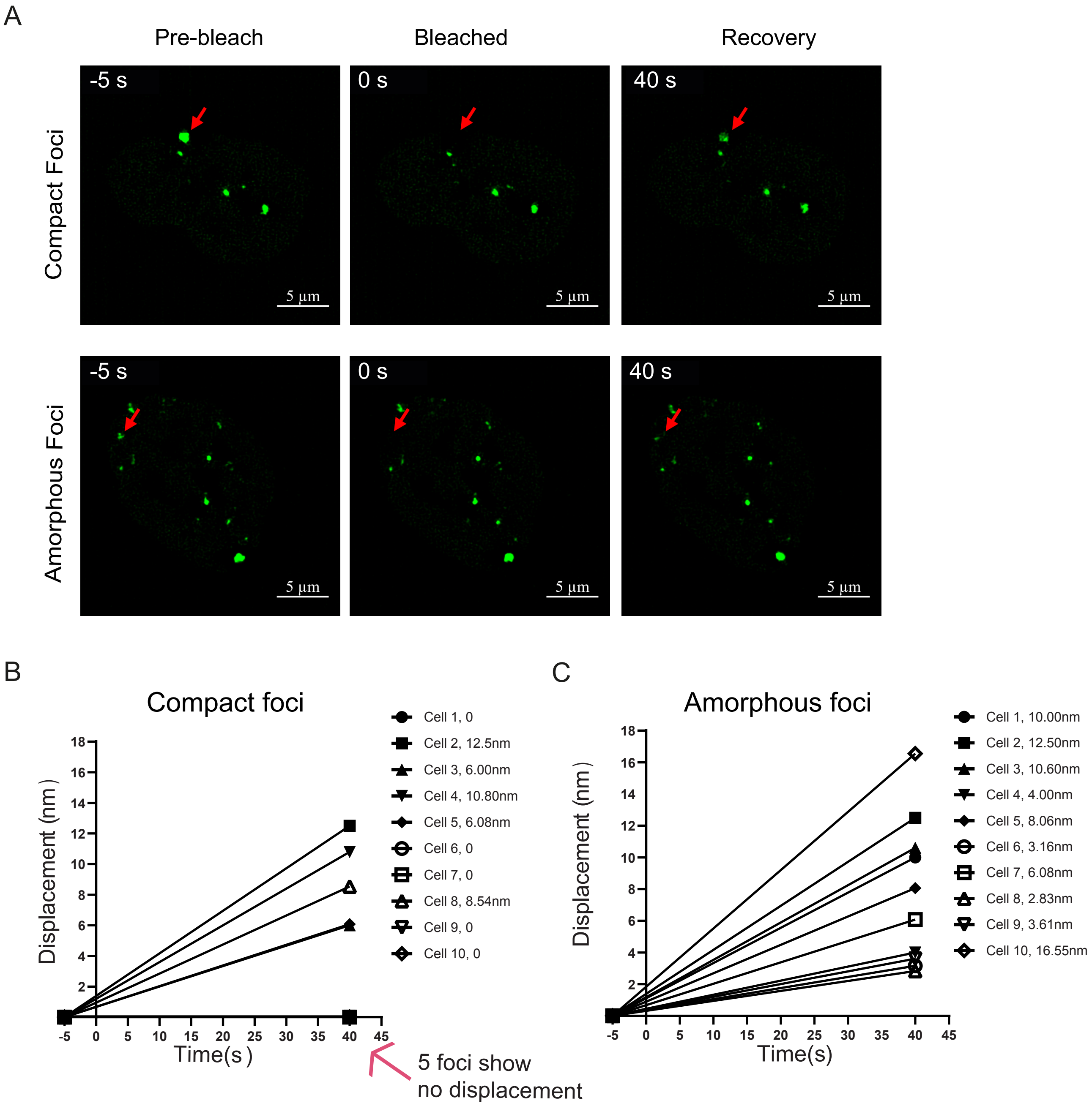


**Figure S3: Compact foci are frequently stationary compared to amorphous 53BP1 foci, related to Figure 4.** **A)** *di*SIM processed super-resolution images of compact and amorphous 53BP1 foci. Representative images from the FRAP study show prebleaching, bleached and recovery images. Red arrows mark the bleached foci. **B)** and **C)** Displacement of centroids of 53BP1 foci between prebleached and recovered status (40 seconds post-bleaching) showed a small but significant increase in the amorphous foci compared to compact foci (n=10 cells, each condition). Pink arrow shows five foci with no discernible displacement. Displacement of all photobleached foci indicated on the right (as legend).

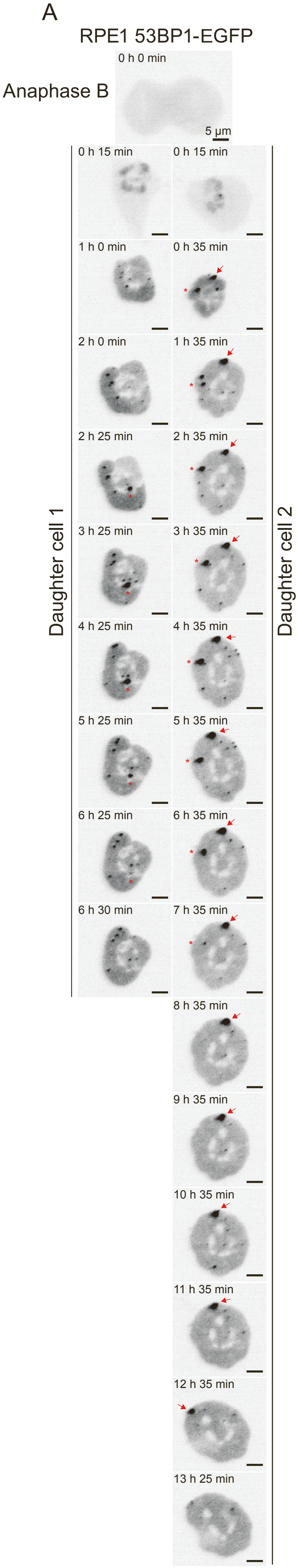


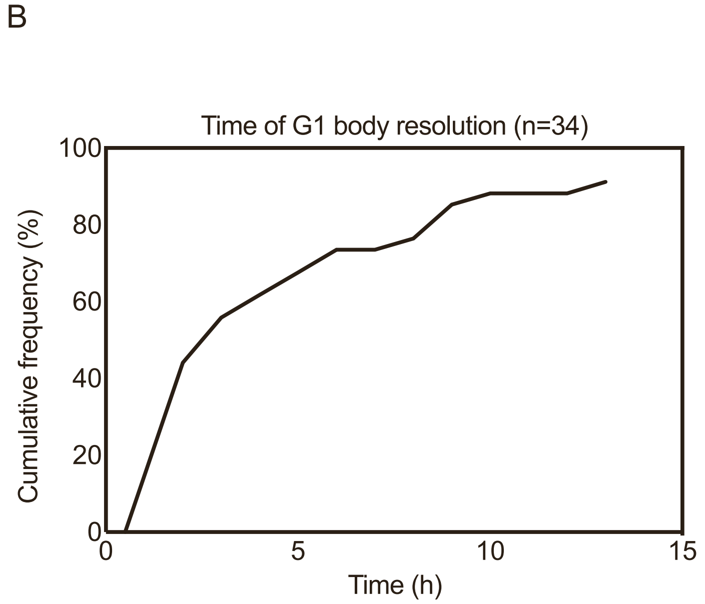


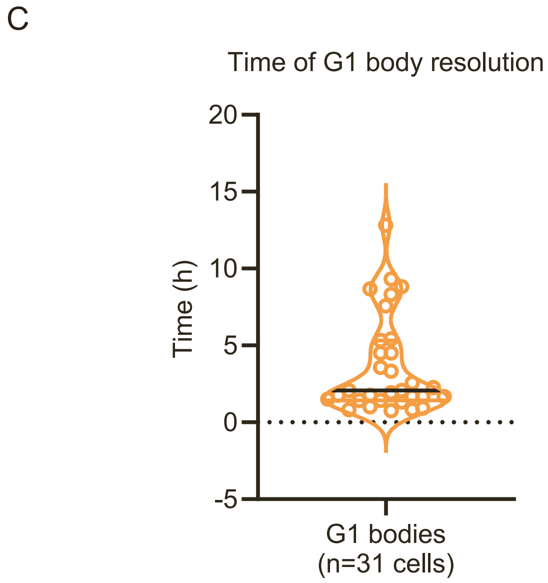


**Figure S4: G1 body resolution time show a highly variable period between 53BP1-EGFP foci appearance and disappearance, related to Figure 5.**
**A)** Cropped Lattice Light-Sheet (LLS) microscopy images show G1 bodies that form soon after mitosis in two daughter cells. RPE1 53BP1-EGFP cells were treated with aphidicolin overnight for 10-16 hours and released before 24 hours of imaging. Red arrows mark the G1 body that remains through the movie with a long resolution time (>10 h), and red asterisks marks G1 bodies with moderate resolution time (3-7 h). Data representative of 4 independent repeats (n = 34 cells). Scale bar as indicated. **B)** Cumulative frequency (%) graph showing the time taken to resolve G1 bodies in time-lapse movies of cells treated as in A. 3 of 34 cells did not resolve foci during the period of imaging. **C)** Violin plot showing median time taken for the resolution of G1 bodies in cells released from aphidicolin treated as in (A). 3 cells that failed to resolve are omitted from this data. Median value is marked using a black line.

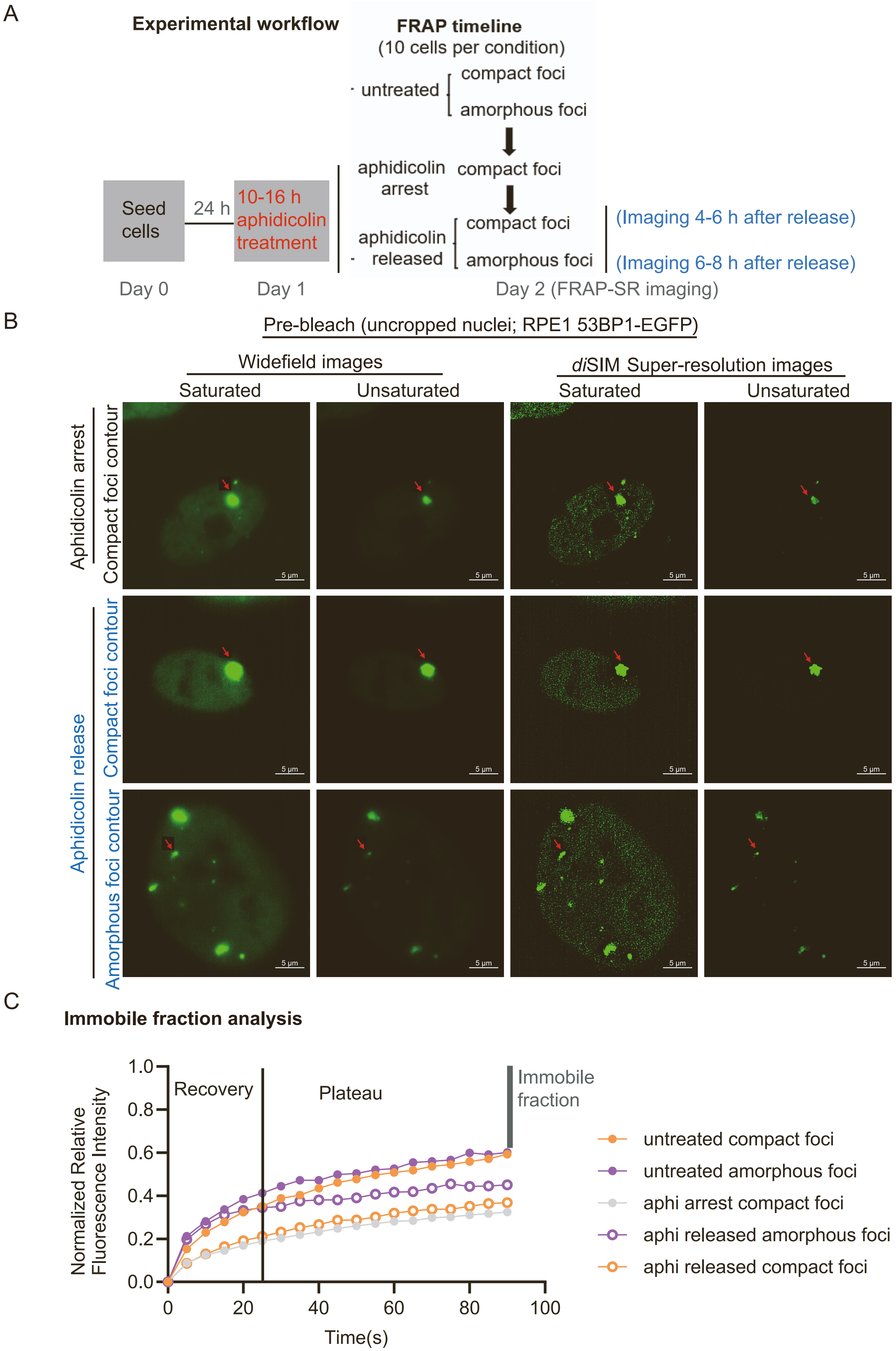


**Figure S5: Aphidicolin synchronisation (arrest-release) shows 53BP1 foci changes across the cell cycle, related to Figure 6.** **A)** Aphidicolin treatment and release regime for live-cell imaging. RPE1 53BP1-EGFP cells treated with aphidicolin overnight for 10-16 hours were released and imaged for compact or amorphous foci as indicated. **B)** Uncropped saturated and unsaturated widefield super-resolved images of cells treated as in A. Prebleach images are presented. Scale bar as indicated. **C)** Graph of normalised mean values of FRAP recovery curves to highlight differences in immobile fraction of 53BP1-EGFP in compact or amorphous foci-bearing cells. T6 (25 s) was used as a threshold based on t1/2 in Figure 6B, 2C and 3C for recovery and plateau period studies in Figure 6E.

Sequence analysis of the targeted and non-targeted allele-specific PCR products are shown below as provided by the vendor (Horizon/Revitty™).

A) PCR sequencing outcomes
Legend: gRNA binding site PAM site

| Parental (hTERT RPE1) |
|---|
| -V--S--Q--E--W--V--I--Q--C--L--I--V--G--E--R--I--G--F--K--Q--H--P--K--Y--K--H--D--Y--V--S--H----------------*- |
| GTGTCACAAGAGTGGGTGATCCAGTGCCTCATTGTTGGGGAGAGAATTGGATTCAAGCAGCATCCAAAATATAAACACGATTATGTTTCTCAC--------------TAAAGATACTTGGTCTTACTGGTTTTATTCCCT |

Clone 213 c-terminal eGFP cassette insertion

| Allele 1 |
|---|
| -V--S--Q--E--W--V--I--Q--C--L--I--V--G--E--R--I--G--F--K--Q--H--P--K--Y--K--H--D--Y--V--S--H----------------*- |
| GTGTCACAAGAGTGGGTGATCCAGTGCCTCATTGTTGGTGAGAGAATTGGATTCAAGCAGCATCCAAAATATAAACACGATTATGTTTCTCAC[C-EGFP cassette]AGATACTTGGTCTTACTGGTTTTATTCCCT |

| Allele 2 |
|---|
| -V--S--Q--E--W--V--I--Q--C--L--I--V--G--E--R--I--G--F--K--Q--H--P--K--Y--K--H--D--Y--V--S--H----------------*- |
| GTGTCACAAGAGTGGGTGATCCAGTGCCTCATTGTTGGTGAGAGAATTGGATTCAAGCAGCATCCAAAATATAAACACGATTATGTTTCTCAC[C-EGFP cassette]AGATACTTGGTCTTACTGGTTTTATTCCCT |

B) Table of PCR primers used to scan edits

| **PCR Amplification Product** | **Forward Primer (5'-3')** | **Reverse Primer (5'-3')** | **Product Size** |
|---|---|---|---|
| Non-targeted allele-specific | AGGTGTTAGGCCTTGTGGTCCA | GCCAGCTGTCCTCCGTAAGTGA | 784 bp |
| Targeted allele-specific (Left homology arm) | CTGGACTGGTAAGTATTTGAAGCCC | TGGACGTAGCCTTCGGGCATGG | 1800 bp |
| Targeted allele-specific (Right homology arm) | GCAACCTCCCCTTCTACGA | GGCCCAGCCATCACTGGTAATC | 1388 bp |
| Insert specific | TTGATGTGGTGGTGACGGACC | GGGGAACTTCCTGACTAGGG | 1029 bp |

**Table S1: Genomic sequence analysis of RPE1 53BP1-EGFP clone 213, related to Figure 1C.**
A) Table of PCR sequencing results shows modifications on each allele for clone 213 hTERT-RPE1 - TP53BP1 (C-EGFP/C-EGFP) following sequence analysis of the targeted and non-targeted allele-specific PCR products. B) Table of PCR primers and products used to scan CRISPR-engineered edits.
